# DNA methylation/hydroxymethylation in melanoma

**DOI:** 10.18632/oncotarget.18293

**Published:** 2017-05-30

**Authors:** Siqi Fu, Haijing Wu, Huiming Zhang, Christine G. Lian, Qianjin Lu

**Affiliations:** ^1^ Department of Dermatology, Second Xiangya Hospital, Central South University, Hunan Key Laboratory of Medical Epigenomics, Changsha, Hunan, China; ^2^ Program in Dermatopathology, Department of Pathology, Brigham and Women's Hospital, Harvard Medical School, Boston, MA, USA

**Keywords:** melanoma, 5-hmC, 5-mC, epigenetic therapy, TET

## Abstract

Melanoma is a malignant tumor of melanocytes and is considered to be the most aggressive cancer among all skin diseases. The pathogenesis of melanoma has not been well documented, which may restrict the research and development of biomarkers and therapies. To date, several genetic and epigenetic factors have been identified as contributing to the development and progression of melanoma. Besides the findings on genetic susceptibilities, the recent progress in epigenetic studies has revealed that loss of the DNA hydroxymethylation mark, 5-hydroxymethylcytosine (5-hmC), along with high levels of DNA methylation at promoter regions of several tumor suppressor genes in melanoma, may serve as biomarkers for melanoma. Moreover, 5-Aza-2′-deoxycytidine, an epigenetic modifier causing DNA demethylation, and ten-eleven translocation family dioxygenase (TET), which catalyzes the generation of 5-hmC, demonstrate therapeutic potential in melanoma treatment. In this review, we will summarize the latest progress in research on DNA methylation/hydroxymethylation in melanoma, and we will discuss and provide insight for epigenetic biomarkers and therapies for melanoma. Particularly, we will discuss the role of DNA hydroxymethylation in melanoma infiltrating immune cells, which may also serve as a potential target for melanoma treatment.

## INTRODUCTION

Melanoma is the most aggressive form of skin cancer, in which metastasis is the most common cause of death in patients. Melanoma commonly arises from cutaneous melanocytes, but it can also occur on mucosal surfaces such as the oral cavity, gastrointestinal sites, and genital mucosa as well as the uveal tract of the eye and leptomeninges. The pathogenesis of melanoma has not been well elucidated; however, several risk factors have been revealed to be associated with melanoma, such as hair color [1], skin phototype, numerous nevi, ultraviolet exposure [2], and family history of melanoma [3]. Therefore, genetics and environmental factor-induced epigenetic alterations have been found to contribute to melanoma. In recent decades, billions of dollars have been invested into the research on genetic susceptibilities to melanoma and development of genetic therapies. Thus far, thousands of mutational events have been observed in the melanoma genome, and the melanoma genome has been revealed to be characterized by high frequencies of mutations carrying a signature of ultraviolet-B radiation [4–5]. *BRAF* is the gene most frequently mutated (50-70%) in melanoma, and *BRAF*^v600E^ is the most common mutation, which is usually found in benign nevi [6]. Despite the advances in gene targeting therapy, the development of inhibitors of mutant *BRAF* kinase, for example, as therapeutic agents, is stagnant due to resistance to the therapy [7]. In addition, variations in DNA sequence alone cannot completely explain the biological differences that separate benign nevi from melanoma. Therefore, increasing attention is being focused on the participation of epigenetic events.

Epigenetics refers to the study of potentially heritable changes in gene expression and function that do not involve alterations of the original nucleotide sequence of DNA. Epigenetic modifications are primarily comprised of DNA methylation, histone modification, and microRNA (miRNA)-mediated and long non-coding RNA (lncRNA)-mediated regulation. These epigenetic mechanisms ultimately determine whether genes are expressed or silenced; therefore, these epigenetic mechanisms play critical roles in various life processes such as cell differentiation, growth, development, aging and immune response [8]. Epigenetics provides an explanation for how environmental factors contribute to our individual phenotype as well as an explanation for susceptibilities to certain diseases such as cancer. In addition, epigenetic status may be more easily manipulated, compared to gene therapies, rendering epigenetic modifications more therapeutically reversible. Therefore, in this review, we will summarize the latest progress made in research on epigenetic modifications, especially DNA methylation/hydroxymethylation, in melanoma, and we will discuss their potential applications as biomarkers and therapeutic strategies for personalized treatment.

## DNA METHYLATION AND HYDROXYMETHYLATION

**Figure 1:**
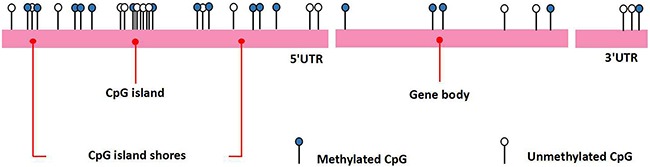
The CpG islands and CpG island shores

**Figure 2:**
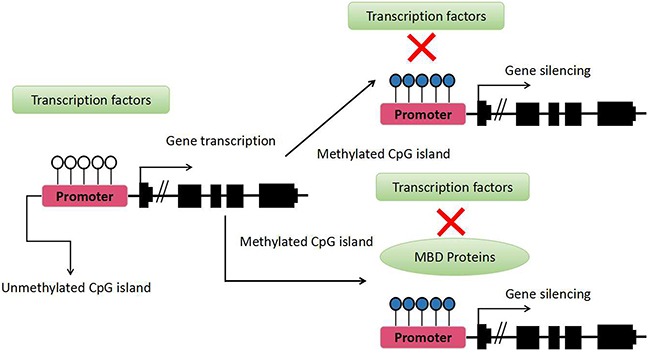
How DNA methylation regulates transcription

DNA methylation is a relatively stable and heritable epigenetic mark in several eukaryotic organisms. It is a biochemical process in which a methyl group is added to a cytosine or adenine at the 5-position on the pyrimidine ring of the methyl group where the DNA base thymine is located, converting cytosine to methylcytosine [9]. The CpG dinucleotides tend to cluster in regions called CpG islands, defined as regions of more than 200 bases with a G + C content of at least 50% and a ratio of observed to statistically expected CpG frequencies of at least 60%. Approximately 60% of gene promoters are associated with CpG islands and are normally unmethylated, although some of them (approximately 6%) become methylated in a tissue-specific manner during early development or in differentiated tissues [10]. This finding may explain why all cells in an organism share the same genetic information, but they show different phenotypes. In general, CpG island methylation is associated with gene silencing. DNA methylation serves as a mark that indicates repression of gene expression; therefore, it is involved in several biological processes, such as cell differentiation and proliferation. DNA methylation inhibits gene expression by various mechanisms. Methyl-CpG-binding domain (MBD) proteins, for example, can be recruited by methylated DNA; in turn, MBD family members recruit histone modifying and chromatin-remodeling complexes to the methylated sites [11]. Moreover, DNA methylation can directly inhibit transcription by precluding the recruitment of DNA-binding proteins to their target sites [12]. However, DNA methylation does not occur exclusively at CpG islands; it may also occur at CpG island shores, which refer to regions of lower densities of CpG that lie close to CpG islands and are associated with transcriptional inactivation (Figure 1). Most tissue-specific DNA methylation occurs not at CpG islands but at CpG island shores [13]. In mammalian cells, DNA methylation is restricted to regions of CpG islands, which are typically present in promoter regions [14] (Figure 2). The process of DNA methylation is mediated by methyltransferases, such as DNMT1, DNMT3a, and DNMT3b, and each of them displays different functional capacities. For example, DNMT1 maintains methylation status during cell replication, whereas DNMT3a and 3b usually induce de novo methylation [15].

**Figure 3:**
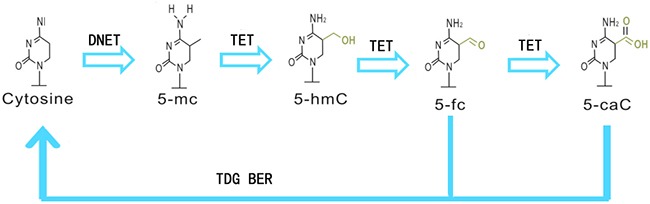
The cycle of DNA methylation and demethylation

In contrast, DNA demethylation is a process that occurs passively, especially by the programmed failure of transmission of methylation patterns during a round of cell division, and re-activates or re-expresses silenced genes [16]. Unlike DNA methylation, DNA demethylation has been less studied, and active DNA demethylation in mammals has been recognized only recently. This process occurs through the sequential iterative oxidation of the methyl group of 5-mC and removal of the final modified group by the actions of thymine DNA glycosylase (TDG) as well as the base excision repair pathway to yield cytosine from 5-mC [16]. Oxidation of 5-mC to 5-hydroxymethylcytosine 5-hmC is the first and most important step of this reaction, which is mediated by the TET family dioxygenase enzymes, including TET1, TET2 and TET3 [17]. 5-hmC is the most abundant intermediate of the active DNA demethylation process and acts as a positive transcriptional regulator in normal development and cancer [18–19], and its levels are directly correlated with the levels of differentiation in a wide variety of human tissues [20]. All three TETs can further oxidize 5-hmC to 5-formylcytosine (5-fC) and 5-carboxylcytosine (5-CaC), resulting in tissue levels in the order of 5-mC>5-hmC>5-fC>5-CaC [21]. Meanwhile, both formylcytosine and carboxylcytosine can be excised by TDG, which triggers subsequent base excision repair (BER), indicating a potential role for active demethylation [19, 22] (Figure 3).

Generally, 5-hmC levels are significantly lower and vary greatly depending on the cell type (0.1-0.7% of total cytosine) compared to the relatively constant levels of 5-mC in somatic tissues (3–4% of total cytosine) [23–24]. The three TET family proteins vary in levels in different cell types. For example, TET1 and TET2 are highly expressed by embryonic stem cells and in early embryogenesis, and their levels decrease when cells exit pluripotency and undergo differentiation. TET2 is highly expressed in the hematopoietic system, while TET3 is overexpressed in germ cells/oocyte, brain tissue and more ubiquitously in somatic cells. TET proteins are responsible for generating all of the 5-hmC in the genome [25], and the loss of TET functions may have dire biological consequences. Numerous loss-of-function mutations of *tet2* have been identified in myeloid cancers where *tet2* has been shown to be a key tumor suppressor. In addition, TET1 has been recently revealed to play an oncogenic role in MLL-rearranged leukemia [26]. Moreover, the loss of 5-hmC has been observed in several malignancies, such as breast cancer [27], liver cancer [28], and kidney cancer [29], and has even been proposed as a prognostic marker in ovarian cancer [30]. However, if TET proteins are capable of oxidizing 5-mC to 5-CaC, the question arises as to why this reaction stops at 5-hmC and why 5-hmC is stable and abundant in the genome. Recent findings of differences among TET proteins suggest that perhaps 5-hmC and other oxidized forms may have epigenetic roles in addition to their function as DNA demethylation intermediates [31–32].

### The mechanism for loss of 5-hmC in cancer

In the past decades, a more comprehensive frame-work of DNA demethylation has been documented and the loss of methylation has been found to occur by various mechanisms: active loss through iterative oxidation of 5-mC to 5-hmC, 5-fC and 5-CaC by TET proteins followed by excision of 5-fC and 5-CaC by TDG [16]; active loss through deamination of 5-mC to U catalyzed by AID and APOBEC1 followed by BER [33]; passive loss whereby methylation is diluted during several division cycles due to lack of maintenance activity by DNMT1 and ubiquitin-like proteins containing PHD and RING finger domains 1 (UHRF1), and a RING finger-associated mammalian SRA (SET- and RING-associated) domain protein that is required to maintain 5-mC in the CG context [34]. UHRF1 SRA specifically recognizes hemi-5-mCG sites [35], which are the products of semi-conservative DNA replication. In addition, by recruiting maintenance DNMT1, UHRF1 SRA facilitates the restoration of hemi-5-mCG to full-5-mCG after each round of DNA replication in mammals [36]. In the current understanding, passive demethylation is the dominant mechanism for demethylation of the genome, combined with active removal of 5-mC by TET proteins and replicative loss of both 5-mC and 5-hmC [37–40]. Loss of 5-hmC in tumors occurs through two mechanisms: inactivating mutations of *Tet* and inhibition of TET activity by isocitrate dehydrogenase 1/2 (IDH1/2) mutations [41–45], and these mutations are common in leukemia rather than in solid cancers. Only one study revealed that *Dnmt1* mutations [46] and the resultant losses of substrates of 5-mC are not key players in tumorigenesis.

### Abnormal 5-mC level and regulated genes in melanoma

In previous studies, global DNA hypomethylation was observed in the neoplastic progression of carcinogenesis [47–48]. One hypothesis is that hypomethylation allows previously neoplastic cells to proliferate and eventually metastasize as well as to exert a survival advantage [49]. Moreover, DNA hypomethylation in or around centromeric repeats and other repetitive sequences has been observed to be associated with chromosomal instability [50]. On the other hand, DNA hypermethylation of CpG islands at promoter sites is believed to contribute to tumorigenesis by silencing tumor suppressor genes 17. Numerous tumor suppressor genes, which are hypermethylated and involved in biological processes, including cell cycle regulation, DNA repair, cell signaling, transcription and apoptosis, have been reported in melanoma (Table 1). Furthermore, the tendency towards hypermethylation has been termed the ‘CpG island methylator phenotype’ (CIMP) [51–52].

**Table 1:** The hypermethylated genes in melanoma

Gene	Relevance to melanoma	Ref
***LINE-1***	Associated with metastasis	[54]
***CLDN11***	Inactivation of tumor related gene	[58–59]
***TERT, MGMT,***	Associated with clinical characteristics	[63]
***KIT, TNF, MITF***	Observed in metastatic melanoma and inhibits invasion in melanoma cells	[92–93]
***RASSF6,***	Related to the pathogenesis of MM	[94]
***RASSF10***	Overexpression of MMP9 in MM	[95]
***GPX3***	Decreased expression in MM	[96]
***MMP-9***	Arrests cell cycle in G1 by inhibiting G1 cyclin-CDK	[97–98]
***SYNPO2***	Inhibition of cell proliferation	[97]
***CDKN1C***		[97]
***LXN***	Unknown	[99]
***WFDC1, SYK,***	Inhibition of tumorigenesis by reducing IKKα/β phosphorylation	
***QPCT, PCSK,***	Promotion of tumor aggressive via TGF-β1-MMP3	[100]
***MFAP2,***	etcReduction of cytokine-induced effects;	[100]
***ASC/PYCARDC/***	Blockade of G1/S and M phase;	[101]
***PYCARD***	Association with CDH1	[102–103]
***Col11A1***	Unknown	
***SOCS1***	Linked to cadmium-stimulated cell growth and inhibition of death pathway	[100]
***ZFYVE28, ZBTB47, etc***	A cell adhesion molecules; loss correlates with high tumor grade and poor prognosis	[100]
***Caspase 8***	Renders cancer cells resistant	[101]
***CDH1***	Tumor suppressor gene	[102–103]
***MGMT***	Acts on IFN-γ pathway	[102]
***RAR-b2***	Attenuates cytokine-induced effects	[102]
***CIITA-PIV***	Decoy receptor that protects cells from TRAIL-mediated apoptosis	[103]
***SOCS2***	Control of actin-mediated cell motility	[103]
***TNFRSF10C (DcR1/2)***	Dominant negative regulator of angiogenesis	[103]
***TPM1***	Arrests cell cycle in G1 by inhibiting CDK4 and CKD6 and activating pR8	[103]
***TIMP3***	Decline in serum from melanoma patients	[103–104]
***CDKN2A***	A metastasis suppressor; inhibits Wnt5a signaling	[105]
***DPPIV***	Inhibits IL-17/Stat3 pathway; suppresses tumor growth in mouse model	[106–107]
***FRZB***	Mediates cell-to-cell and cell-to-matrix interactions which is important for platelet aggregation and angiogenesis	[108–109]
***SOCS3***	Downregulation associated with transformation and progression	[110–111]
***THBS1***	Mediates cell-to-cell and cell-to-matrix interactions which is important for platelet aggregation and angiogenesis	[112]
***TM***	Downregulation associated with transformation and progression	[113]

Apart from CDKN2A, RAR-b2, RASSF1A and IDH1, which have been intensively discussed in other reviews [49, 53], the hypermethylation of other genes has also been linked to melanoma. LINE-1, for example, a transposable element that has been used as a surrogate marker for global methylation in several cancer studies, has been found to be hypermethylated in Brazilian melanoma patients and is suggested to be a biomarker for cutaneous melanoma [54]. LINE-1 methylation status is reported to be associated with cancer risk, whereby both the hypermethylation and hypomethylation status appear to vary between different cancer types [55–57]. In addition, it has been suggested that *Claudin 11 (CLDN11)* could be a useful epigenetic biomarker for identifying melanoma [58–59]. The Claudin gene family consists of 27 members, which encode membrane proteins of the paracellular tight junction. The locations of metastases have been observed to be significantly correlated with the methylation frequency, indicating that the methylation levels in primary melanoma may contribute to differences in the metastatic capacity of melanomas. It would be interesting to analyze the functional alteration by CLDN11 inactivation in greater detail. Moreover, the methylation status of *MGMT*, which encodes a repair protein by removing alkyl groups from the O6-position of guanine residues and its promoter, has been proposed as a biomarker in glioblastoma [60–61], colorectal cancer [62] and melanoma [63]. In melanoma, epigenetic silencing of this gene has been demonstrated in tumors and serum of patients [63–65], suggesting an important role of *MGMT* in tumor development. *MITF* is another example of a DNA hypermethylated gene, which is a transcription factor that controls cell cycle and melanogenesis genes [66–67]. The hypermethylation of the *MITF* promoter has been reported in peripheral blood of melanoma patients who develop more than one lesion, and the *MITF* gene has been observed to be hypermethylated in primary tumors compared to metastatic tumors. Interestingly, the expression of *MITF* varies intratumorally and among different melanoma specimens [68], with high expressions being associated with active differentiation or proliferation and relatively low expressions indicating invasion capacity [69]; these findings suggest that high methylation levels at the *MITF* promoter might be associated with a more aggressive disease and that the higher methylation levels at the *MITF* gene of primary tumors compared to metastatic tumors have a role in controlling the cell cycle. However, more studies are needed to further clarify the functional alterations and roles of hypermethylated genes observed in melanoma.

### Abnormal 5-hmC level and regulated genes in melanoma

As the global hypomethylation marker within the bulk of the genome, the loss of 5-hmC has been observed and used as a biomarker to distinguish melanomas from physiological melanocytes and benign melanocytic proliferations [70]. In the same study, a strong correlation between the loss of 5-hmC and poor prognosis in melanoma has been identified, suggesting 5-hmC level as a potential biomarker with predictive value. Other studies, conducted in the subsequent years, have confirmed this finding [71–75]. Based on this phenomenon, a principle question arises as to the loss of 5-hmC being the cause or consequence of melanoma. Another question is regarding the unknown mechanism of the loss of 5-hmC in melanoma. Although reduced levels of IDH2 and TET proteins have been observed in melanoma, the upstream modulation and consequences of this alteration are still unclear. Targeting TET proteins has been suggested to be a therapeutic strategy in cancer [76]. However, due to the overall hypomethylation levels in melanoma, it is unclear whether TET proteins may act as a cure or a killer.

Notably, the studies mentioned above are focused on the 5-hmC levels in the whole melanoma skin lesion, rather than specifically in melanocytes, which means the tumor infiltrating immune cells may also be included. In our previous study, the infiltrating CD8+ and CD4+ T cells were 20-50% of the total cells under the microscope, and the 5-hmC levels were lost in the T cells (unpublished data). As is well known, T cells play a critical role in the anti-tumor immune responses. Furthermore, it is a well-accepted notion that cancer cells, such as malignant melanocytes express high levels of PD-L1, which can help cancer cells escape from the PD-1-expressing T cells [77–83]. The questions arise as to whether there is a relationship between high levels of PD-1 and loss of 5-hmC in T cells and whether the loss of 5-hmC contributes to the reduced cytotoxicity of CD8+ T cells in melanoma. Indeed, loss of 5-hmC in the promoter region of *Pdcd-1* has been reported to contribute to the lasting PD-1 expression in T cells in a peptide immunotherapy (PIT) mouse model [84], suggesting a possible role of 5-hmC in PD-1 expression. However, *Pdcd-1* is just one example of a melanoma-related gene; little is known about the involvement of other genes, such as perforins, which are DNA methylation-sensitive genes and have been observed to be hypomethylated in autoimmune diseases in our previous studies [85–86]. In addition, high levels of 5-hmC have been observed in lupus T cells [87], which are the hyper-activated T cells in contrast to tumor infiltrating T cells [8]. Therefore, further investigation into the epigenetic modifications of tumor infiltrating T cells may shed light on the pathogenesis of melanoma and other cancers and provide novel therapeutic strategies.

### Epigenetic biomarkers and therapies in melanoma

As mentioned above, the methylation levels in the promoters of *LINE-1*, *TERT, MGMT, KIT, TNF, MITF* [54, 63], among others*.,* and especially the loss of 5-hmC by immunohistochemistry, have the potential to be used as biomarkers to aid in distinguishing malignant melanocytic lesions from dysplastic or borderline melanocytic lesions [70]. The loss of 5-hmC provides a simple and fast method for diagnosis, and it has been recapitulated in other human tumors.

With the reversible advantage, several epigenetic therapies have been approved by the FDA. Azaciticine (Vidaza^TM^) and Decitabine (Dacogen®), which are the DNMT-1 inhibitors, have been approved for treating myelodysplastic syndromes; the drugs are still in clinical trial for melanoma treatment [49]. However, the sole use of DNMT inhibitors in melanoma treatment has yielded mixed results, which might be due to the heterogenicity of tumors and the overall epigenetic alterations. Moreover, reduced expression of TET2 in melanoma may provide novel options [88–89]. As discussed above, the DNA methylation status has been observed to be low in the neoplastic progression of carcinogenesis. Therefore, gene specific modification should be considered for future application. As the Crisper-Cas9 technique is in its blooming era, genetic and epigenetic therapy may benefit from this gene editing revolution. Furthermore, the DNA methylation levels in tumor infiltrating T cells may also provide novel strategies for treatments.

## PERSPECTIVES

Despite the developments in chemotherapy and genetic therapies, poor prognosis of metastatic melanoma and increasing incidence of this malignant disease demands novel and quick strategies for earlier diagnosis and personalized therapies with higher efficacy. In addition to DNA methylation, other epigenetic modifications, such as histone modification and non-coding RNAs, as well as the interplay of these modifications, should be intensively studied for a better understanding of the pathogenesis of melanoma. The varying levels of microRNAs have been observed to be one of the consequences of DNA methylation in cancers [90–91]. Epigenetic alterations may also promote genetic mutations and genomic rearrangements in cancer, though the underlying mechanisms remain unclear. The tumor microenvironment, which might contribute to the unique phenomenon of loss of 5-hmC in melanoma, should be investigated further to improve the current understanding, which may have immense translational implications for benefiting patients afflicted with advanced melanoma and other cancers.
